# Participatory health research under COVID-19 restrictions in Bauchi State, Nigeria: Feasibility of cellular teleconferencing for virtual discussions with community groups in a low-resource setting

**DOI:** 10.1177/20552076211070386

**Published:** 2022-01-04

**Authors:** Khalid Omer, Umaira Ansari, Amar Aziz, Khalid Hassan, Lami Aminati Bgeidam, Muhd Chadi Baba, Yagana Gidado, Neil Andersson, Anne Cockcroft

**Affiliations:** 1 Centro de Investigación de Enfermedades Tropicales (CIET), 341132 Universidad Autónoma de Guerrero, Acapulco, Mexico; 2 Federation of Muslim Women Association of Nigeria (FOMWAN), Bauchi, Nigeria; 3 Department of Family Medicine, CIET-PRAM, 5620 McGill University, Montreal, Canada

**Keywords:** Participatory research, communities, focus groups, teleconference, cellular networks, low- and middle-income countries, COVID-19

## Abstract

**Introduction:**

During the COVID-19 pandemic, researchers have used Internet-based applications to conduct virtual group meetings, but this is not feasible in low-resource settings. In a community health research project in Bauchi State, Nigeria, COVID-19 restrictions precluded planned face-to-face meetings with community groups. We tested the feasibility of using cellular teleconferencing for these meetings.

**Methods:**

In an initial exercise, we used cellular teleconferencing to conduct six male and six female community focus group discussions. Informed by this experience, we conducted cellular teleconferences with 10 male and 10 female groups of community leaders, in different communities, to discuss progress with previously formulated action plans. Ahead of each teleconference call, a call coordinator contacted individual participants to seek consent and confirm availability. The coordinator connected the facilitator, the reporter, and the participants on each conference call, and audio-recorded the call. Each call lasted less than 1 h. Field notes and debriefing meetings with field teams supported the assessment of feasibility of the teleconference meetings.

**Results:**

Cellular teleconferencing was feasible and inexpensive. Using multiple handsets at the base allowed more participants in a call**.** Guidelines for facilitators and participants developed after the initial meetings were helpful, as were reminder calls ahead of the meeting. Connecting women participants was challenging. Facilitators needed extra practice to support group interactions without eye contact and body language signals.

**Conclusions:**

With careful preparation and training, cellular teleconferencing can be a feasible and inexpensive method of conducting group discussions in a low-resource setting.

## Introduction

Social distancing restrictions imposed during the COVID-19 pandemic have prevented face-to-face meetings with groups of research participants for qualitative data collection and accelerated the adoption of technology for conducting such meetings remotely.^1,2^ Participatory research engages stakeholders, including patients, community members, and others, throughout the research process.^3^ It can involve quantitative and qualitative methods; a key feature is that it usually involves talking to groups of stakeholders, for example, in focus group discussions or deliberative dialogues.^4,5^ Holding such group discussions can be challenging. Even in non-pandemic times, in low- and middle-income countries, it can be difficult and expensive to hold face-to-face discussions in remote communities or to bring people from such communities to a central point for a group discussion.

The authors have reported increased use of Internet-based technologies, including video conferencing, to conduct virtual meetings, mainly in higher-income countries.^6–13^ Participants need access to equipment such as laptops or smartphones, as well as an Internet connection. Many people in low- and middle-income countries, especially in rural communities, have neither. Telephone conferencing methods predate Internet-based methods and can be an effective way of conducting focus group discussions, especially for geographically dispersed participants.^14–17^ The published reports about telephonic meetings are from higher-income countries; we are not aware of reports of using simple telephone-based technology to conduct remote community group discussions in low- and middle-income countries.

In Bauchi State, Nigeria, we conducted a participatory mixed-method research project to implement and test the impact of universal home visits on maternal and child health in Toro Local Government Area (LGA).^18^ The home visits took place between 2016 and 2019. In early 2020, we met face to face with groups of community leaders to present and discuss with them the quantitative research findings about impact of the home visits^19–21^ and plan with them follow-up actions they could implement in their communities. In May and June 2020, we intended to hold follow-up meetings with community leaders to discuss their progress with action plans. The Federal Government of Nigeria imposed COVID-19 restrictions from the end of March 2020.^22,23^ The social distancing requirements and local travel restrictions precluded face-to-face meetings in communities. The study we describe in this paper aimed to test the feasibility of using cellular teleconferencing to conduct virtual meetings with community stakeholder groups in Toro LGA, Bauchi State, Nigeria, a low-resource setting in a lower-middle-income country.

## Methods

### Setting

Bauchi State is in north-eastern Nigeria. Based on projections from the 2006 national census, it has an estimated population of around 5 million. Toro is the largest LGA in Bauchi, with a projected population of 487,100 living in 18 wards (administrative areas, with a population of 10,000–100,000). Cellular network coverage in Nigeria has grown rapidly over the last decade; there are currently more than 184 million subscribers nationwide, with a teledensity of around 97%.^24^ Internet coverage is much lower, with about 65 million subscribers and a population penetration of 32%.^25^ We chose to use teleconferencing to support virtual community meetings because Bauchi state has good coverage with cellular networks but the Internet connectivity is patchy (28%).^25^ Also, few cellular network users have smartphones, especially among women.

### First round of teleconferencing

Since the disruption caused by COVID-19 was a topic of great interest and concern in mid-2020, we used this as the theme for the first round of telephonic community group discussions. We created a discussion guide about COVID-19 and its prevention and trained two teams (one male and one female) to conduct telephonic community focus group discussions. Each team comprised a facilitator, a reporter, and a call coordinator. The discussion guide asked participants about their knowledge and perceptions about COVID-19, causes for its spread, and precautions people could take individually and as a community to avoid the infection. As part of their training, the trainees convened two practice cellular teleconferences, with other members of the research team as participants.

We selected six communities, one from each ward included in the home visits project, to give a spread of urban, rural, and rural–remote communities. In these communities, we conducted 12 focus group discussions (6 female and 6 male groups) with community members. The focal person in each ward (assigned by the LGA to coordinate primary health care activities in the ward) provided a list of men and women potentially available to join the group discussions in each community, with their cell phone contact numbers. After identifying the best cellular network to use for each community, the call coordinator made individual calls to participants, to explain the purpose of the conference call, seek their consent to participate, and confirm the time and date of the conference call.

**Figure 1.**
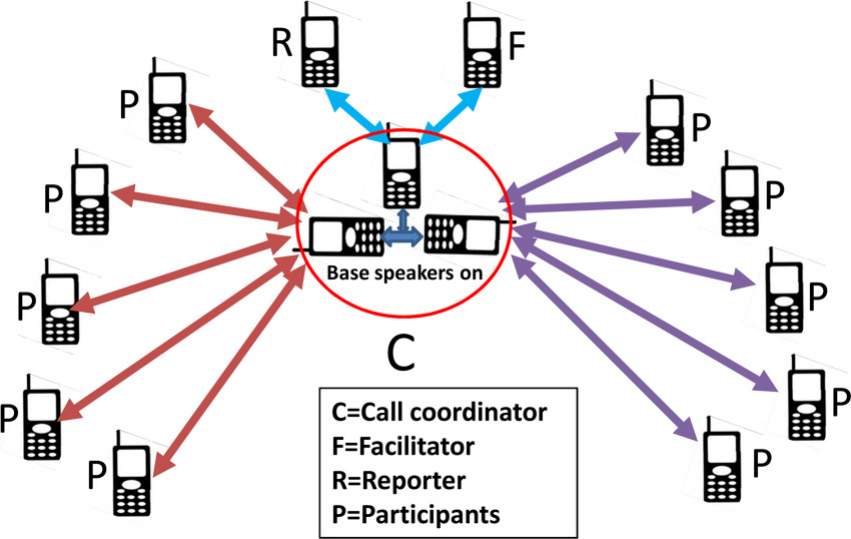
Diagrammatic representation of a teleconference using three handsets.

We recruited 8–10 participants for each group. The cellular conference call facility only allows a maximum of six participants to connect from a single base phone. With two or sometimes three team members (facilitator, reporter, and supervisor) connected on the call, this meant only three or four community members could participate. To increase the number of participants possible in the group calls, we used two or three handsets with their speakers on at the base and distributed community participants into simultaneous calls from these handsets (Figure 1).

At the scheduled time, the call coordinator made a cellular conference call to connect the facilitator, the reporter, and the participants. Each meeting lasted for about an hour and we audio-recorded the discussion with the participants’ consent. We asked the participants not to disclose who else attended the meeting and not to repeat anything that was said outside the group. Participants could receive the call on basic handsets, but the call coordinator initiated the call using an Android handset to monitor that everyone stayed connected and to record the discussion. The reporter took notes of the discussion during the call. The reporter and facilitator sat together soon after the meeting and used the notes, the audio recording, and their recall to prepare a final report. A supervisor from the research team attended some meetings to observe the dynamics and conduct of the discussion. She or he provided constructive feedback to the team. Based on the experience of this first round of teleconferencing, we developed checklists for the call coordinator and for participants (see Box 1).

### Second round of cellular teleconferencing

These teleconference meetings with groups of community leaders followed on from the previous face-to-face meetings with the same groups, in which the field teams had shared and discussed the findings about impact of the

Box 1: Checklist for coordination and conduct of teleconference

**Guidelines for call coordinator**

- Coordinate with ward focal person to get the list of potential participants
- Call individual participants to explain about the discussion
- Check availability; time, and date
- Finalize the call date and time based on availability of participants
- Call individual participants again to confirm the availability for final date and time
- Ensure calling handsets have enough airtime balance to complete the call
- Ensure handsets are charged and working properly
- Ensure recording function is working well
- Call each participant 1 h before the meeting to remind them and explain guidelines
- Charge handset before the call
- Check signals and choose a quiet place
- Monitor if all the participants remain connected; call again if anyone gets disconnected

**Guidelines for participants**

- Ensure cellular handset is available nearby
- Charge handset before the call
- Check signals and choose a quiet place
- Identify yourself before speaking
- Please respect one another's privacy by not discussing who attended this meeting or repeating anything that was said
- Avoid cross talk during the call
- Send an SMS to the call coordinator if the quality of sound is bad

home visits and supported the groups to develop plans for community activities to sustain improvements due to the visits. The short discussion guide for the conference call included questions about progress with the planned community activities and any barriers and constraints faced. We conducted 20 group meetings (10 males and 10 females) with community leaders in 10 communities (including urban, rural, and rural–remote communities). The communities did not include any that had participated in the first round of teleconference meetings. Three community leaders participated in each 30–40 min meeting.

The teams conducted the meetings according to the guidelines developed after the first round of telephonic meetings. The facilitator, reporter, and the participants all had a copy of the action plan from the previous meeting (given to the participants at the end of the previous meeting). As with the first-round meetings, we audio-recorded the discussion, the reporter took notes, and the reporter and facilitator worked together after the meeting to prepare a final report.

### Assessment of feasibility

The field teams documented the process of the telephonic meetings in their field reports, including their observations of any difficulties encountered. In a follow-up meeting, the senior research team discussed with the field teams their views and experiences of conducting the telephonic meetings; they documented what worked well and what did not work well.

### Ethical approval

The Bauchi State Health Research Ethics Committee approved the overall project on 12 May 2015 (NREC/12/05/2015/12). The McGill Faculty of Medicine IRB provided approval on 23 June 2015 (A06-B35-15A). The teams conducting the meetings sought verbal informed consent from each participant.

## Results

The 12 first-round telephonic meetings had an average of 8 participants for men's groups and 6 participants for women's groups and lasted about 54 min for men's groups and about 42 min for women's groups (Table 1).

**Table 1.** Cellular teleconference community meetings discussing COVID-19.

Ward	Type of community	Number of participants		Duration (minutes)	
		Male	Female	Male	Female
1	Urban	9	5	48	32
2	Urban	6	6	58	37
3	Rural	6	6	52	45
4	Rural	8	6	54	52
5	Rural	8	6	55	48
6	Rural–remote	8	6	58	38

Three community leaders participated in each of the 20 telephonic meetings in the second round; the women's groups lasted a mean of 45 min, and the men's groups lasted a mean of 31 min (Table 2).

**Table 2.** Cellular teleconference community meetings with community leaders.

Community type	Number of groups	Participants per group	Meeting duration (minutes)
Male groups			
Urban	3	3	30
Rural	6	3	37
Rural–remote	1	3	26
Mean		3	31
Female groups			
Urban	3	3	48
Rural	6	3	46
Rural–remote	1	2	41
Mean		3	45

The teleconference meetings generally went well. The teams faced some difficulties in recruiting and engaging women. Many of the proposed women participants did not have their own handsets and had to borrow handsets from their spouses or other family members. They agreed to participate and told the call coordinator that they would have a phone available, but at the start time of the meeting, they did not have a handset with them. This led to some delays in starting the meetings while a handset was obtained, or a proposed participant was replaced by a woman who had access to a handset.

In the first-round meetings, when we used two base handsets to allow more participants in the conference call (Figure 1), the sound quality remained good. Sometimes, one or two participants dropped out of meetings temporarily because of network connection problems. The call coordinator monitored the connection of each individual participant and was able to reconnect quickly to any that dropped out.

Challenges, especially during the first-round meetings, included participants forgetting the date and time of the call, or forgetting to charge their handsets before the meeting and running out of power during the meeting. In some of the first-round meetings, there were distractions such as participants conducting side discussions, or interference from other family members, or because participants were in a noisy area. Guidelines for the facilitators and participants (see Box 1) helped to overcome these challenges in the second-round meetings with community leaders. Audio recording the discussion worked well because everyone spoke on the telephone. The audio recording of each meeting supplemented the notes taken by the reporter when preparing the final report of the meeting.

The average airtime cost of each call of about 60 min was NGN 2000 (USD 5.26) per handset with five people connected: slightly more than 1 USD/person/meeting. The cost was less when the call coordinator used the same network as most of the participants. All the costs were borne by the project. Cellular networks in Nigeria do not charge subscribers for incoming calls.

The teams reported that they found the telephone groups easier to control than face-to-face meetings; participants were polite and spoke one at a time. But they noted that lack of eye contact with and among the participants and limited group interactions made facilitation of the group and reporting on group dynamics difficult. They believed the participating community members felt comfortable with the cellular teleconferencing as a modality for their meetings. It was easier for them to participate from their homes or a place convenient for them.

## Discussion

Cellular teleconferencing helped us to conduct meetings with community groups as part of a participatory health project during the COVID-19 pandemic with associated restrictions on travel and face-to-face meetings. This worked well in a low-resource setting where remote meetings using Internet-based technologies are not a feasible option, due to poor Internet connectivity and low ownership of smartphones.

### Advantages

The conference calling saved costs; the only costs were for airtime and for stipends for the field teams. Other authors have reported on the cost-saving associated with conducting focus group meetings remotely.^12,26,27^ Unlike with Internet-based options, with cellular calls, there is no cost to the participants. Based on our experience of face-to-face group meetings in communities in the same area, the cost for travel to each community is around NGN 20,000 (USD 53). Typically, two group discussions are conducted in each community (one male and one female group). The airtime cost of two 1-hour teleconferences was about NGN 4000 (USD 10.52), less than one-fifth of the travel cost.

Teleconferencing also saved the time of field teams traveling to communities. Studies in high-income countries have reported that telephone focus groups are useful to bring together participants from widespread locations, without participants needing to travel.^15,16,28,29^

In line with other reports,^14^ the facilitators in our study reported that the telephone group discussions were easier to control than face-to-face meetings, perhaps because people are used to taking turns when speaking on the phone one to one. Another potential advantage of group discussions on the phone or online (without video) is that participants feel more anonymous than in a face-to-face meeting and may discuss sensitive topics more openly.^26,28–30^ This was not an issue in our study, because the participants were from the same community and usually already known to one another.

### Challenges

In telephone conference calls, it is difficult to control the environment of the individual participants and ensure they are well prepared for the meeting. Especially during the first-round meetings, our field teams noted problems such as side conversations, intrusion of family members, and noisy backgrounds, and some participants did not charge their handsets before the call. Other authors have reported similar difficulties and highlighted the need for pre-meeting contacts to secure and maintain participation.^9,17^ The guidelines we developed for facilitators and participants and pre-meeting reminder calls helped to overcome these difficulties.

Particularly in low-resource settings, a poor network connection can disrupt telephonic meetings.^17^ In our study, this did not pose a major challenge because the cellular network was reasonably good in the communities included and the call coordinator selected the best network for each community. In some settings, this could be a major challenge.

The limitation on the number of participants for cellular teleconferencing could be a problem, but we demonstrated that this can be readily overcome by using multiple handsets at the base. Other authors have concluded that telephone focus groups in any case work better with fewer participants.^9,31^

Interactions within the group are an important feature of focus group discussions.^30,32^ Our facilitators found it difficult to monitor group dynamics and encourage group interactions over the telephone, and others have reported similar problems.^8,17^ Facilitators for teleconference group meetings need to learn and practice ways of creating a conducive environment for open discussion and ensuring everyone who wants to contribute can do so, without the benefit of eye contact and other body languages.^9^ This requires careful and intensive training and practice for facilitators.^14^

In our study, the facilitators noted that the participation of group members was better in the meetings with community leaders than in the community meetings discussing COVID-19. This might have been partly because of the extra experience of the facilitators by the time of the meetings with community leaders, but we think it was also because the later groups were smaller, and the participants had material about the discussion (the action plans from the previous face-to-face meeting) ahead of the telephone meeting. Other authors have suggested providing questions in advance or sending other material to participants before the meeting to help the discussion.^14^

Remote meetings might exclude people who cannot participate because of lack of network coverage or lack of equipment.^15^ This is more of a concern with Internet-based applications.^33^ In Bauchi, we found that some women were unable to participate because they did not have their own handsets and borrowing a handset from the spouse or other family member was unreliable. Lack of cell phones among women in Nigeria can limit their participation individually and in group discussions. One study in Nigeria using cell phone messaging about breastfeeding found that providing a phone shared between five and seven women, who subsequently discussed the messages, was effective.^34^

### Limitations

We conducted the cellular teleconference meetings in only a small number of communities with relatively good cellular network coverage. We did not ask community participants directly about their experience with the teleconference meetings; we relied on the field team reports that participants seemed comfortable and participated freely in the discussions.

## Conclusion

The COVID-19 pandemic with its associated restrictions has accelerated testing of new ways to conduct community group discussions as part of participatory research. With careful preparation and training, cellular teleconferencing can be a feasible and inexpensive method of conducting group discussions in a low-resource setting.
